# Calcitriol Imparts Neuroprotection *In Vitro* to Midbrain Dopaminergic Neurons by Upregulating GDNF Expression

**DOI:** 10.1371/journal.pone.0062040

**Published:** 2013-04-23

**Authors:** Rowan P. Orme, Manminder S. Bhangal, Rosemary A. Fricker

**Affiliations:** 1 Institute for Science and Technology in Medicine and Department of Life Sciences, Keele University, Keele, Staffordshire, England; 2 School of Medicine, Keele University, Keele, Staffordshire, England; University of Minnesota, United States of America

## Abstract

During development a tightly controlled signaling cascade dictates the differentiation, maturation and survival of developing neurons. Understanding this signaling mechanism is important for developing therapies for neurodegenerative illnesses. In previous work we have sought to understand the complex signaling pathways responsible for the development of midbrain dopamine neurons using a proteomic approach. One protein we have identified as being expressed in developing midbrain tissue is the vitamin D receptor. Therefore we investigated the effect of the biologically active vitamin D_3_ metabolite, calcitriol, on primary fetal ventral mesencephalic cultures of dopamine neurons. We observed a dose responsive increase in numbers of rat primary dopamine neurons when calcitriol was added to culture media. Western blot data showed that calcitriol upregulated the expression of glial derived neurotrophic factor (GDNF). Blocking GDNF signaling could prevent calcitriol’s ability to increase numbers of dopamine neurons. An apoptosis assay and cell birth dating experiment revealed that calcitriol increases the number of dopamine neurons through neuroprotection and not increased differentiation. This could have implications for future neuroprotective PD therapies.

## Introduction

Midbrain dopamine (mDA) neurons are of particular importance owing to their selective degeneration in Parkinson’s disease (PD). Drug therapy is the most common intervention for PD, however current therapies only provide effective symptomatic control for approximately 10 years and do not alter the course of the illness. For this reason, alternative therapies are required that can halt the progression of the disease or even reverse the degenerative process. Future therapies could include neuroprotective strategies, to prevent continued degeneration of the nigro-striatal mDA neurons, or cell replacement therapies using mDA neurons derived from stem cells. In both cases, understanding key extracellular signals that increase the development or survival of mDA neurons may improve the potential of PD therapies.

Neurotrophic factors, such as glial derived neurotrophic factor (GDNF) [1], brain derived neurotrophic factor (BDNF) [2] and mesencephalic astrocyte derived neurotrophic factor (MANF) [3] all confer neuroprotection to midbrain dopamine neurons. GDNF has long been known to protect dopamine neurons in animal models of PD [1] and has previously demonstrated restoration of the nigro-striatal circuit in the brains of MPTP-lesioned monkeys when delivered by infusion [4] or lentiviral transfection [5]. Indeed, GDNF has been used in clinical trials, with variable success, showing improvement in PD symptoms in some cases [6], [7], but limited efficacy in others [8]; possibly because the GDNF was not reaching the target neurons in the substantia nigra and putamen [8]. Therefore, neuroprotection by neurotrophic factors is still considered a plausible therapy for PD although current methods require direct intraparenchymal infusion of these factors because they are not able to cross the blood brain barrier (BBB) [6], [7]. Proteins or signaling molecules that can pass the BBB to elevate expression of these neurotrophic factors or to stimulate neuroprotection themselves are therefore highly desirable.

To identify signaling proteins responsible for specification, maintenance, axonal guidance and survival of developing mDA neurons, we have previously performed multiplexed quantitative proteomic analysis of the developing rat embryonic midbrain [9]. This work identified a number of candidate signaling proteins that may be involved in the development of midbrain dopamine neurons. One protein identified in this study was the vitamin D receptor (VDR). This is a nuclear receptor protein, recognizing the biologically active metabolite of vitamin D_3_, calcitriol. It is already known that vitamin A (retinoic acid) and vitamin C (ascorbic acid) play major roles in the development of mDA neurons [10], [11], so, in the work presented here we sought to investigate similar functions for calcitriol.

While vitamin D is most commonly associated with the growth and remodeling of bone, recent studies have identified a much broader spectrum of activity along with widespread expression of the VDR protein in over 36 different cell types [12] including C6 glioma cells [13] and human glioblastoma cells [14]. Vitamin D_3_ (calcitriol)’s effects are mediated through binding of this ligand to its nuclear receptor and formation of heterodimers with the retinoid receptors, RXR and RAR, and interaction with other coactivator and corepressor nuclear receptors, which are capable of initiating expression of over 500 genes [15]. By regulating this large number of genes and through post-transcriptional modification events, calcitriol has been shown to influence cell proliferation and differentiation[16]–[19]. Importantly for this study, calcitriol has also been shown to increase glial-derived neurotrophic factor (GDNF) expression in the striatum [20] and cortex [21] of adult rats and may also partially protect against 6-hydroxydopamine induced lesions [22]. GDNF is a well-known neurotrophic factor for midbrain dopamine neurons and has been shown to stimulate differentiation of stem cells towards a dopaminergic cell fate [23]. In animal models of PD, GDNF has also been shown to simulate sprouting of remaining axons [4], [5]. Given these pro-dopaminergic effects of GDNF and the possible link with calcitriol, we investigated the effect of calcitriol on cultures of E12 primary rat ventral mesencephalic (VM) tissue.

The results described in this article show that the vitamin D receptor protein is expressed in E12 VM tissue and that calcitriol causes a dose-responsive increase in the number of DA neurons in primary E12 VM cultures. This increase in DA neurons was concurrent with a two-fold increase in GDNF expression. Furthermore, blocking GDNF signaling using heparinase III demonstrated that the increase in DA neurons was a result of calcitriol-induced increase in GDNF expression in cultures. Finally, we show that the increase in DA neurons is due to reduced neuronal death and not increased differentiation of the dopaminergic population. Therefore, vitamin D_3_ and specifically its active metabolite calcitriol have an important role to play in the protection of dopamine neurons, with implications for potential neuroprotective strategies or in neuronal replacement therapies for PD.

## Results

### Vitamin D_3_ Receptor Protein is Expressed in the Developing Rat Ventral Mesencephalon

**Figure 1:**
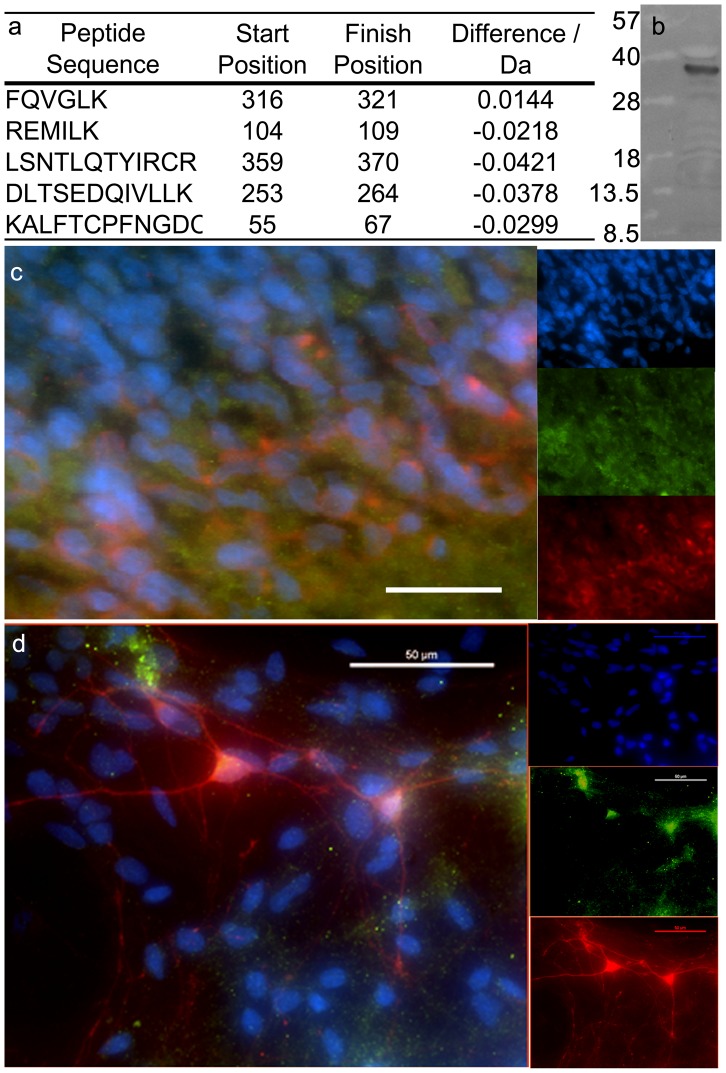
Expression on vitamin D receptor protein. Vitamin D receptor protein was identified in developing ventral midbrain tissue harvested from embryonic rats. A) Five unique peptides were identified and used to identify VDR. B) VDR protein was also identified by a single band in Western blots of whole tissue lysate obtained from E12 VM of rats. C) Immunohistochemistry of E13 sagittal sections taken through the midbrain show overlapping expression of vitamin D receptor (VDR, green) and tyrosine hydroxylase (TH, red). Scale bar: 20 µM. D) Co-expression of VDR and TH was also observed in single cell cultures of E12 VM tissue. Total cells were labeled with DAPI (blue). Scale bar: 50 µM.

We initially identified expression of the VDR protein in the developing rat ventral mesencephalon from a previous multiplexed proteomic investigation into developing midbrain tissue [9]. We profiled proteins expressed in the VM of Sprague-Dawley rats aged E11 to E14 and VDR was identified by mass spectrometry from five unique peptides with a confidence interval greater than 99% (Figure 1A). In addition to the identification of the receptor, we also identified vitamin D binding protein, responsible for transporting calcitriol to target organs, from 4 unique peptides, with a confidence interval of 99.9%. Quantitative data obtained from proteomics suggested there was no difference in the expression of VDR over the time course of VM development studied (tissue ages E11– E14; data not shown).

Western blotting of whole tissue lysates obtained from E12 embryos detected a single band at approximately 34 kDa, corresponding to VDR (Figure 1B). Additionally, 12 µM cryostat sections obtained from E13 embryos were immunolabeled and expression of VDR was observed in the dopaminergic region of the ventral mesencephalon (Figure 1C). Increasing dorso-ventral expression of VDR was observed, showing highest expression at the ventral-most edge of the mesencephalon, corresponding to the dopaminergic region. Monolayer cultures of E12 neurons, dissociated and cultured for 7 days, were also stained for TH and VDR (Figure 1D). Of the TH+ neurons, 94% ±5.4% co-expressed VDR.

These results conclusively demonstrated expression of VDR in the developing midbrain and suggested that vitamin D could play a signaling role during specification of mDA neurons.

### Addition of Calcitriol Increases the Number of Dopamine Neurons in Primary Cultures of Embryonic Rat VM

We next investigated whether the vitamin D_3_ metabolite has any influence on the number of dopamine neurons obtained from primary neural cultures of E12 rat VM tissue. In all experiments, calcitriol was used examine the effects of vitamin D_3_ signaling on neurons as it is the active metabolite produced from the biologically inactive vitamin by two oxidation reactions occurring in the liver and kidneys [12]. To ascertain the optimum concentration of calcitriol, we used varying concentrations added to culture media, ranging from physiological levels (estimated at approximately 100 pM [12]) to 100 nM.

**Figure 2:**
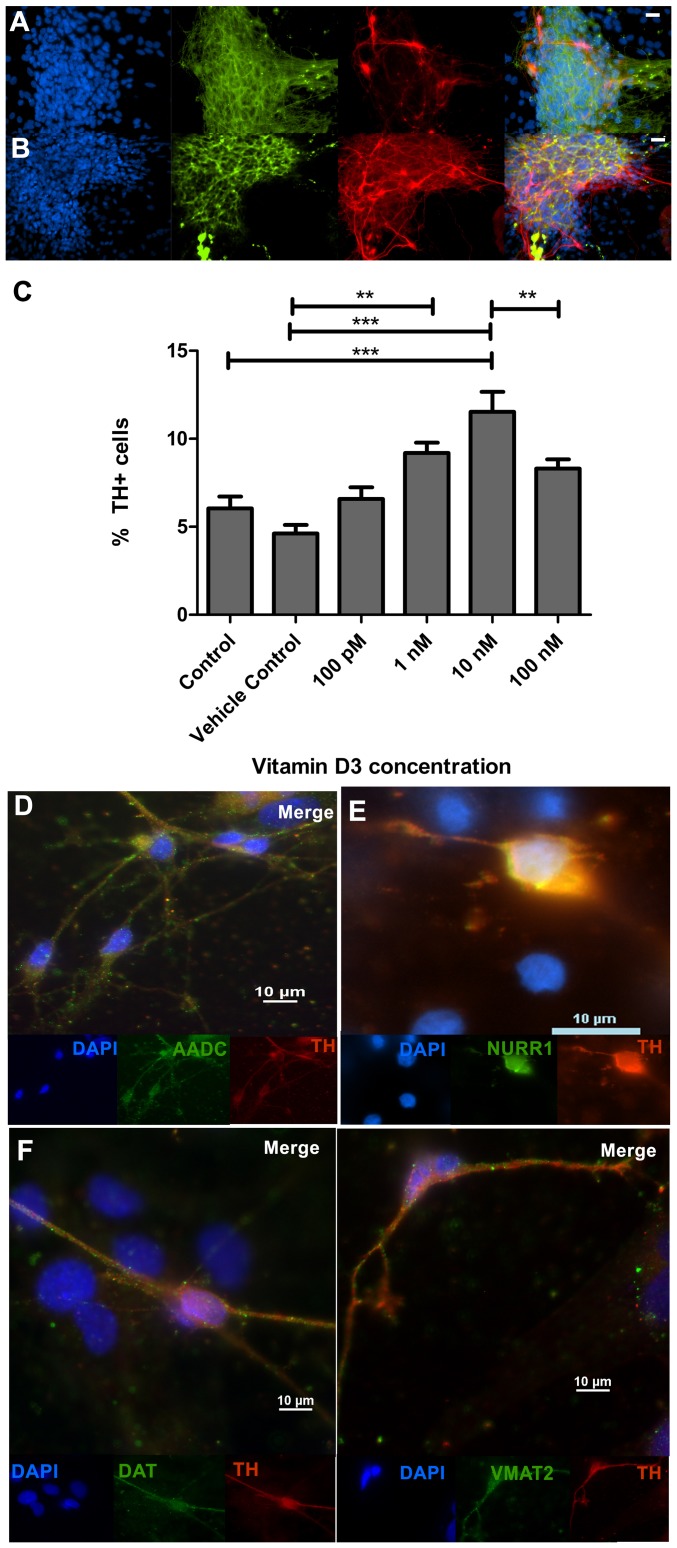
Effect of calcitriol addition to culture media on dopamine neurons. Dose responsive increase in the number of TH immunoreactive neurons was obtained with addition of calcitriol to media. A) primary E12 control cultures and B) cultures with 10 nM calcitriol showing immunostaining with antibodies specific to tyrosine hydroxylase (red) and Tuj1 (green) counterstained with DAPI (blue). Scale bars: 20 µM. C) The percentage of dopamine neurons obtained from cultures with various concentrations of calcitriol added to media. Error bars represent SEM, ***p<0.001, **p<0.01, *p<0.05. The percentage of dopamine neurons obtained from primary E12 ventral mesencephalic cultures increased when calcitriol was added to media up to an optimum concentration of 10 nM. Dopamine neurons were also shown to be immunoreactive for other ventral midbrain DA neuron marker proteins: (D) aromatic acid decarboxylase (AADC); (E) Nurr1; (F) dopamine transporter (DAT); and (G) vesicular monoamine transporter 2 (VMAT2). Scale bars: 10 µM.

Following seven days of culture, calcitriol elicited a concentration dependent increase in the percentage of tyrosine hydroxylase immunoreactive (TH+) neurons (ANOVA (*F*_(5,498)_ = 13.58, p<0.0001). At 10 nM calcitriol concentration, the percentage of TH+ neurons of the whole cell population was increased by almost two fold compared to control conditions (6.0% ±0.7% in control cultures, vs. 11.5% ±1.1% with 10 nM calcitriol, Bonferroni post-test t = 6.14, p<0.01) (Figure 2 A–C).This was shown to be solely an effect of calcitriol addition as no significant difference was observed between control and vehicle only conditions (5.74% ±0.5 vs. 4.7% ±0.6%, Bonferroni post-test t = 0.86, n.s.). In cultures exposed to 100 nM calcitriol, the percentage of TH+ neurons was not significantly different to levels obtained under control conditions. There was no difference in the percentage of β-III tubulin+ cells between control and 10 nM calcitriol-stimulated conditions (41.5% ±2.4 vs. 36.2% ±2.6, unpaired t-test t = 1.49, n.s.). The ventral midbrain dopaminergic identity of the TH+ neurons was confirmed through co-expression with other DA markers: aromatic acid decarboxylase (AADC), the nuclear orphan receptor Nurr1, the dopamine transporter (DAT), and vesicular monoamine transporter 2 (VMAT2) (Figure 2 D–G).

### GDNF Expression is Upregulated by Calcitriol

**Figure 3:**
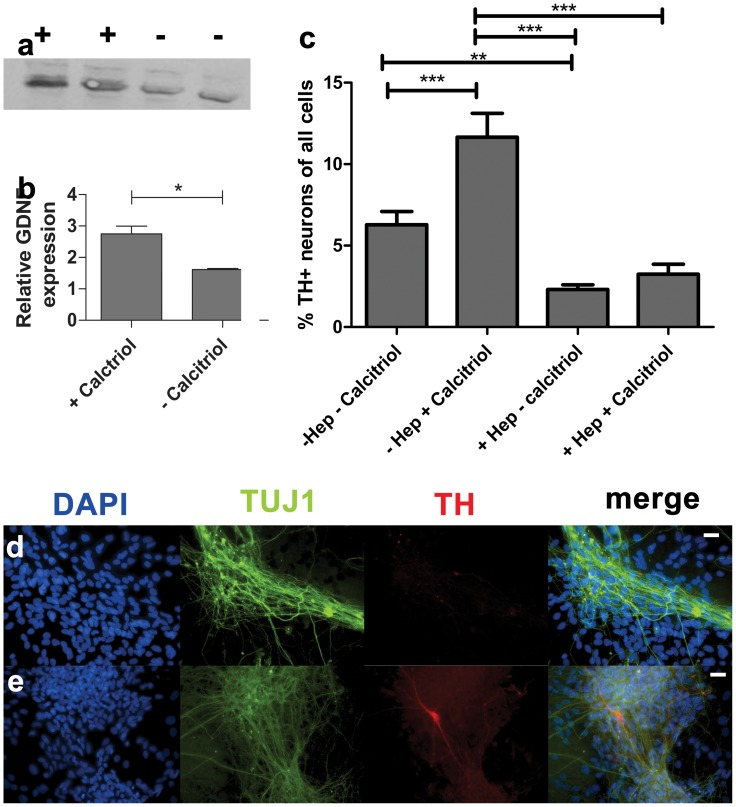
Relationship between calcitriol and GDNF signaling. The beneficial effect of calcitriol addition to media was shown to be a result of increased GDNF. (A, B) 10 nM calcitriol added to media caused almost a 1.8-fold increase in GDNF expression, observed by Western blotting. GAPDH was used as loading control; lanes:+with calcitriol, - control; *p<0.05. In the presence of 0.3 U heparinase III, the number of dopamine neurons observed was significantly decreased regardless of calcitriol incorporation (C). Bars represent percentage tyrosine hydroxylase immunoreactive neurons as a percentage of total cells±SEM. ***p<0.001, **p<0.01. Representative images of primary ventral mesencephalic cultures supplemented with 0.3 U heparinase III without calcitriol (D) and with the addition of 10 nM calcitriol (E). Cultures were stained with tyrosine hydroxylase (red) and β-III tubulin (green). Scale bar: 20 µm.

GNDF is a neurotrophic factor that is known to influence development and survival of dopamine neurons. As GDNF has previously been shown to be upregulated in response to calcitriol stimulation [13], [20], [21], we used Western blotting to see if expression of GDNF was also upregulated in our cultures. Primary E12 VM cultures were cultured either in media containing 10 nM calcitriol or control media (no calcitriol) for 7 days. Total protein was extracted from the cultures and transferred to nitrocellulose membrane for detection with an anti-GDNF antibody. Using integrated optical density measurements normalized to GAPDH expression, GDNF expression was increased 1.8±0.2 fold (n = 7, unpaired t-test, t = 4.4, p<0.05) in cells treated with 10 nM calcitriol compared to control cultures (Figure 3A, B).

### Increased Numbers of Dopamine Neurons are Directly Attributable to Calcitriol-induced Increase in GDNF Expression

GDNF signaling relies on heparin sulphate proteoglycan binding, and therefore treatment with Heparinase III will inhibit exogenous GDNF signaling [24], [25]. To determine whether or not GDNF expressed in response to calcitriol is responsible for the increase in dopamine neurons obtained from E12 VM tissue, we blocked GDNF signaling through the addition of 0.3 U/ml Heparinase III to cultures.

Blocking GDNF signaling had a significant effect in reducing the percentage of dopamine neurons in E12 VM cultures (ANOVA F_(3,167)_ = 22.05, *p*<0.0001). In cultures without calcitriol, heparinase III reduced the percentage of dopamine neurons of the total cell number by approximately three fold in comparison to control media (2.3±0.3% in +Hep cultures vs. 6.3±0.8% in control cultures, Bonferroni post-test *t* = 3.21, *p*<0.001) (Figure 3C). This is likely a result of inhibiting GDNF that is naturally expressed in cultures. The reduction in DA neurons increased to five-fold when compared to numbers of DA neurons observed in cultures containing 10 nM calcitriol but no heparinase III (2.3±0.3% in +Hep cultures vs. 11.7±1.5% in +Calcitriol cultures, Bonferroni post-test *t* = 7.39, *p<*0.01).

To see if heparinase III could prevent the effect of calcitriol-induced increase in DA neurons, we exposed E12 VM cultures to 10 nM calcitriol and 0.3 U/ml heparinase III simultaneously for 7 days. Under these conditions, calcitriol failed to counteract the effects of heparinase III and there was no difference in the numbers of DA neurons between cultures treated with calcitriol+heparinase III and cultures with heparinase III alone (2.3±0.3% vs. 3.2±0.6%, Bonferroni post-test *t* = 0.75, *p = *0.17) (Figure 3C–E). This suggests that calcitriol is exerting a neuroprotective effect through the elevation of GDNF signaling.

### Calcitriol Increases Dopamine Neurons in Culture through a Reduction in Apoptosis

**Figure 4:**
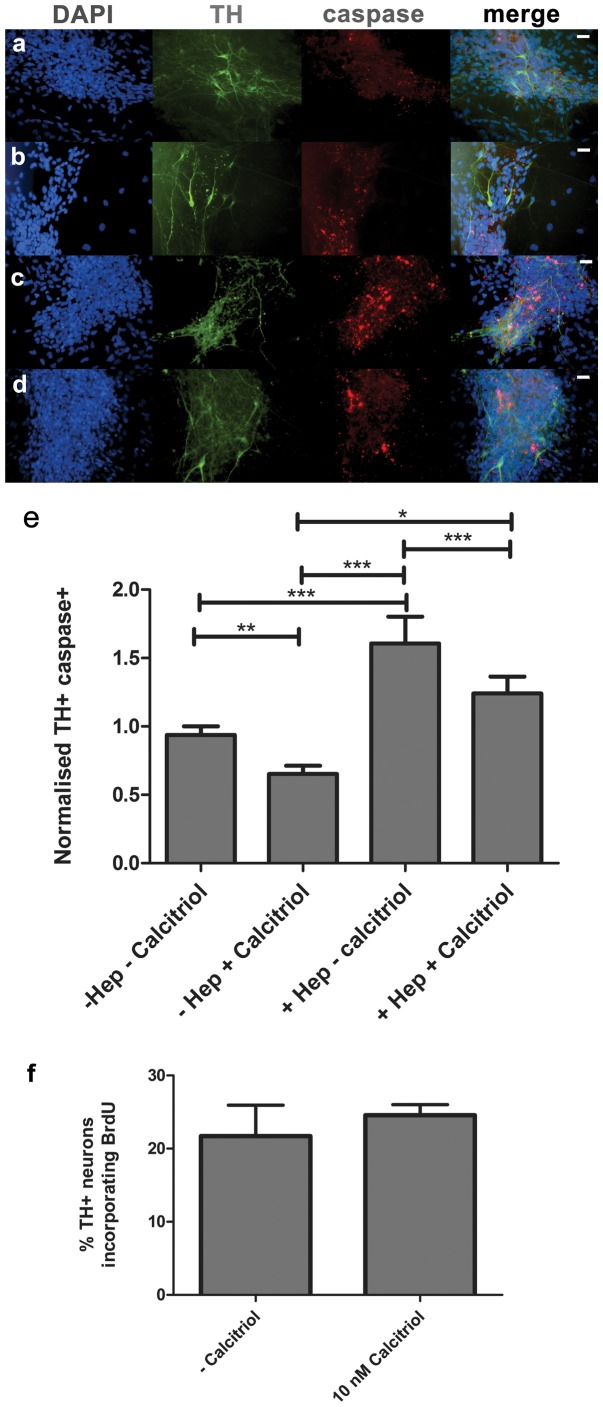
Calcitriol is neuroprotective and does not increase differentiation. The increase in dopamine neurons obtained from cultures was shown to be a result of increased neuroprotection afforded through calcitriol addition, rather than changes in differentiation. (A–D) Immunolabeling with a pan-caspase marker (red) and antibodies to tyrosine hydroxylase (green) counterstained with DAPI (blue), for: A) control media, B) 10 nM calcitriol, C) 0.3 U/ml Heparinase III and D) 10 nM calcitriol with 0.3 U/ml heparinase III. Scale bars: 20 µM. E) The number of apoptotic dopamine neurons was significantly increased when heparinase III was added to media. Bars represent the ratio of caspase+ dopamine neurons to caspase+ DAPI+ cells. Error bars represent SEM, ***p<0.001, *p<0.05. F) The percentage of TH+ neurons that had undergone terminal mitotic division and specification during culture was not significantly different when 10 nM calcitriol was added to cultures. Bars represent percent BrdU+ dopamine neurons, error bars are SEM.

To identify the mechanism by which calcitriol-mediated GDNF increases the number of dopamine neurons obtained from primary VM cultures, we performed a caspase assay to identify the number of apoptotic cells in culture. A pan-caspase kit was used to label cells at all stages of apoptosis. To highlight the dopamine neuron-specific effects, the proportion of caspase-positive DA neurons was normalized against the proportion of caspase-positive cells in the total cell population (where a ratio of 1.0 indicates that dopamine neurons are equally as susceptible to cell death as all cells in the culture). Our data indicates that calcitriol increases the number of DA neurons in culture through a reduction in apoptosis (ANOVA *F_(3,182)_* = 15.04, *p<*0.0001) (Figure 4 A–E).

In the normalized data we observed a significant decrease in the amount of apoptotic dopamine neurons when calcitriol was added, compared to control media (0.93±0.06 vs. 0.65±0.06, Bonferroni post-test *t* = 2.12, *p<*0.05) (Figure 4 A, B, E). However, when 0.3 U heparinase III was added to media, a significant increase in the ratio of apoptotic dopamine neurons was observed in comparison to media containing 10 nM calcitriol (1.61±0.2 in +Hep cultures vs. 0.65±0.1 in+calcitriol cultures, Bonferroni post-test *t* = 6.38, *p*<0.001) (Figure 4 B, C, E). This was partially reversed in media containing both 0.3 U/ml heparinase III and 10 nM calcitriol (1.24±0.1 vs. calcitriol alone, Bonferroni post-test *t* = 4.34, *p*<0.001) (Figure 4 D, E). Calcitriol was able to reduce the proportion of DA neurons undergoing apoptosis in the presence of heparinase III, (1.24±0.1 in +Hep +calcitriol cultures vs. 1.61±0.2 in +Hep cultures, p<0.001) (Figure 4 C, D, E).

### Calcitriol does not Increase Dopaminergic Differentiation in Primary Neural Cultures

To confirm whether the increase in dopamine neurons was a result of neuroprotection or an increase in differentiation of neural progenitors to a DA neuron phenotype, 10 µM bromodeoxyuridine (BrdU) was added to the media during the first three days of culture. BrdU is a synthetic thymidine analog that is incorporated into mitotic cells. Cells that had divided in the presence of BrdU were then identified through antibody staining, and cells double-labeled with BrdU and TH were classed as DA neurons that were newly differentiated in culture following terminal mitotic division. There was no difference in the number of double-labeled BrdU+/TH+ neurons comparing cultures containing calcitriol and controls, Figure 4F (24.6% ±1.5 vs. 21.7% ±4.25, unpaired t test, *t* = 0.63, *p = *0.54 ). This demonstrates that calcitriol has no direct effect on differentiation, therefore any changes in DA neuron numbers are related to neuroprotection.

## Discussion

Neurotrophic factors play a crucial role in neuroprotection, both during development and in the adult brain. Using a dose response assay we have demonstrated that calcitriol can upregulate GDNF expression in primary neural cultures and increase the numbers of DA neurons obtained. Through GDNF blocking studies we showed that increases in DA neurons were in response to increased GDNF signaling and were due to neuroprotection rather than increased differentiation. Taken together, this data suggests that calcitriol could be used in future neuroprotective strategies against the progression of PD.

E12 is a critical time point in the development of mDA neurons. We have shown here, through a combination of proteomics, immunohistochemistry and Western blotting that the VDR protein is expressed in the rat VM specifically at that time, corroborating previous evidence that the vitamin D receptor is expressed in the developing rat brain [26]. Importantly, the vitamin D binding protein, which is responsible for transporting vitamin D_3_ and its metabolites to target organs [27], was also identified by proteomic screening of the developing ventral mesencephalon [9]. Coupled with the facts that biologically active forms of vitamin A (retinoic acid) and vitamin C (ascorbic acid) have already been shown to play a major role in the development of the midbrain dopamine neurons our data here suggest that vitamins and their metabolites may have an essential role to play in the development of the midbrain.

Using a dose response investigation, it was clear that addition of 10 nM calcitriol for a culture period of 7 days significantly increased the number of dopamine neurons. At this optimum concentration of calcitriol we observed almost a two fold increase in the number of TH+ neurons obtained from E12 VM cultures. This represents a considerably higher concentration than the physiological level of circulating vitamin D_3_, which is thought to be in the range of 50–125 pM [12]. It is important to consider however, that the amount of calcitriol added to cultures may not accurately reflect the bioavailable calcitriol bound to serum proteins, therefore a direct comparison between physiological levels and culture levels may not be appropriate.

In accordance with other studies [13], [20], [21], we showed that GDNF protein levels were increased approximately two fold in response to calcitriol addition. Furthermore, through the addition of 0.3 U/ml heparinase III to cultures, we demonstrated that the increase in dopamine neurons was a direct consequence of elevated GDNF levels. Addition of GDNF to primary dopaminergic cultures has been long acknowledged to promote survival of dopamine neurons and reduce apoptosis [28]. GDNF signaling requires heparin sulphate glycosaminoglycans [24] and is therefore greatly attenuated by addition of heparinase III to cultures [25], as binding of GDNF to heparin is essential to allow the neuroprotective effects of GDNF [29].In the absence of GDNF signaling dopamine neurons became significantly more prone to apoptosis than the cell population as a whole, as determined by pan caspase expression. This is in accordance with the known neuroprotective properties of GDNF on midbrain dopamine neurons.

GDNF has been used in clinical trials of PD in an attempt to slow dopamine neurodegeneration. Open label trials have shown benefits of continuous infusion of GDNF into the putamen of PD patients [6], [7], however these have not been supported by double blind investigations [8], [30] and several side effects have been reported [5]. It has been suggested that direct delivery into the putamen results in uneven distribution of GDNF resulting in limited bioavailability [31]. Systemic administration of GDNF is likely to be unsuccessful owing to the limited penetration into brain tissue from the blood stream [6]. However, passage of GDNF through the blood-brain barrier using molecular Trojan horse technology, did demonstrate protection against a chemically induced lesion of the nigro-striatal pathway [32]. Small molecules are able to cross the blood brain barrier, therefore systemically administered calcitriol may be able provide better neuroprotection through elevating GDNF expression within the basal ganglia without the need for intraparenchymal infusion. Importantly, expression of the vitamin D receptor protein has been described in the adult human brain, with robust expression observed in the substantia nigra [33]. Alternatively, direct delivery of calcitriol to the putamen through continuous perfusion could also be considered.

It has been postulated that vitamin D deficiency may play a role in the degeneration of DA neurons in PD patients. There is evidence to suggest that polymorphisms in the VDR gene confer susceptibility to PD [34], [35]. Additionally, low serum levels of vitamin D are associated with PD [36], [37]. Coupled with the observation that VDR expression is higher in the substantia nigra than other brain regions [33], these facts indicate that dopamine neurons could be more susceptible to alterations in vitamin D signaling. Seemingly in contradiction to these data, vitamin D restriction in *klotho* mice rescues degeneration of the mesencephalic dopamine neurons. However, this could indicate that *klotho* mice are unable to regulate vitamin D signaling normally, leading to high serum levels which in turn can induce susceptible neurons to undergo cell death [38]. With these points in mind, any use of vitamin D as a therapy must be carefully controlled to avoid excessive serum concentrations. The strong links between vitamin D signaling and PD however, coupled with our observations of neuroprotection detailed in this manuscript suggest that a future therapy focused on the control of vitamin D signaling is a possibility.

To conclude, this work supports previous evidence that gene expression can be directly stimulated by small, biologically active molecules, including vitamins and their metabolites. We have shown conclusively that the VDR is expressed in developing rat ventral midbrain. Furthermore, the bioactive vitamin D_3_ metabolite calcitriol can increase the numbers of dopamine neurons obtained from primary neuronal cultures of the developing ventral midbrain. This effect is a direct result of upregulation of GDNF protein levels and subsequent neuroprotection, and not an effect of increased differentiation. These important findings indicate that vitamin D_3_, and its biologically active metabolite calcitriol, could have important roles to play in future neuroprotective or neurorestorative PD therapies. To investigate the potential of this work to the fullest, future studies should concentrate on *in vivo* analysis to ascertain whether or not calcitriol, administered systemically, can improve survival of dopamine neurons in animal models of PD or in grafted cells.

## Materials and Methods

### Ethics Statement

All animal experiments were conducted in accordance with the UK Home Office Animals (Scientific Procedures) Act 1986. Sprague-Dawley rats used for the study were bred and housed in a dedicated on-site facility and provided with food and water *ad libitum*.

### Animals

Time mated dams were sacrificed by cervical dislocation at embryonic day (E) E12 or E13 (day of plugging designated as E0). The uterine horns were removed, the embryonic sacs opened and embryos removed, killed using schedule one methods, and transferred to ice cold Dulbecco’s modified Eagle’s medium (DMEM) containing 0.6% glucose, 0.12% sodium bicarbonate, and 50 mM HEPES buffer. Tissue and dissecting media were maintained at 4°C to minimize protein degradation.

### Proteomic Analysis

The proteomic analysis of tissue has been described in detail in a previous publication [9]. Briefly, ventral mesencephalic tissue was obtained from embryonic rats aged E11 to E14 and proteins extracted. Tryptic peptides were produced by incubation of reduced and alkylated proteins in trypsin for 24 hours at 37°C. Peptides were labeled with iTRAQ reagents, combined and lyophilised. Labeled peptides were dissolved in 2.4 ml of 10 mM phosphate, 20% acetonitrile and resolved by strong cation exchange. Resulting fractions were then separated using reverse phase chromatography and spotted onto target plates for mass spectrometric analysis. Samples were analyzed by tandem matrix assisted laser desorption ionization mass spectrometry and proteins identified using GPS explorer.

### Preparation of Embryonic Sections

Embryos were removed as previously described and fixed in 4% PFA for twelve hours before equilibrating in a 30% sucrose solution until they sank. Fixed embryos were embedded in OCT media and frozen sections cut at a thickness of 12 µm using a cryostat. Serial sections were collected on subbed microscope slides and allowed to dry.

### Primary Neuron Culture

E12 tissue was prepared as described and incubated for 30 minutes in 0.05% DNase, 0.1% trypsin in DMEM. Tissue was washed in 0.05% DNase and a single cell suspension produced by gentle trituration using a P200 pipette tip. Cells were pelleted and re-suspended in primary culture media (Neurobasal (Invitrogen) containing 1% antibiotic-antimycotic (PAA), 1% v/v B27 (Invitrogen), 1 mM L-glutamine (Sigma), 0.45% w/v glucose (Sigma), 1% v/v fetal calf serum (Sigma)). Viable cells were counted using trypan blue exclusion and a final cell density of 1,000 live cells/µL produced by dilution in primary culture media. 30,000 cells were dropped into the center of a poly-d-lysine and laminin coated coverslip and incubated for 4 hours to allow attachment. Wells were then flooded with 500 µL of culture media, with addition of an extra 500 µL every second day. For studies using vitamin D_3_, the biologically active metabolite, calcitriol (1,25–dihydroxy vitamin D_3_; 1,25(OH)_2_D_3_), was added to media. Heparinase III (R&D Systems) was used to block GDNF signaling [24], [25], [29] by adding 0.3 U/ml to culture media. Following seven days of culture, cells were washed in phosphate buffered saline and fixed using 4% paraformaldehyde at 4°C for 30 minutes.

For BrdU assays, primary neurons were prepared as described and cultured in media containing 10 µM BrdU for the first three days of culture. Media was then removed and cells washed before adding fresh media without BrdU. Additional media was added every second day for a total of 7 days culture.

### Immunochemistry

Sagittal sections or cultured cells were briefly rinsed in TRIS buffered saline (TBS) before blocking in TBS containing 5% normal goat serum for 1 hour. Primary antibodies were diluted in TBS and applied to slides overnight at room temperature for sections or 4°C for cells at the following concentrations: rabbit anti VDR (Abcam) 1∶750; mouse anti tyrosine hydroxylase (Chemicon); rabbit anti tyrosine hydroxylase (Chemicon) 1∶1,000; rabbit anti GDNF (Abcam) 1∶1,000; mouse anti β-III tubulin (Covance) 1∶500. Sections or cells were then washed three times for 5 minutes each in TBS and incubated in fluorescent secondary antibodies (Cheshire Sciences) for 2 hours at 1∶300. Stained samples were washed three times in TBS for 5 minutes each, before rinsing in deionized water and cover-slipping using hard set mounting media containing DAPI (Vector Labs). Images were collected using a Nikon T80i inverted fluorescent microscope using DAPI, FITC and TRITC filters fitted with a Hamamatsu Orca imaging system controlled by NIS Elements.

### Western Blotting

For Western blotting of embryonic ventral midbrain, tissue was dissected out and placed in ice cold lysis buffer (6 M urea, 2 M thiourea, 4% 3-[(3-Cholamidopropyl) dimethylammonio]-1-propanesulfone (CHAPS), 0.5% sodium dodecyl sulphate (SDS)). Tissue from all embryos taken from a single dam was pooled together and broken up by sonication. Samples were stored at −80°C until required. Alternatively, cultured cells were washed with PBS and detached by addition of trypsin for 1 minute. Cells were harvested by addition of 10x volume of culture media and centrifugation at 1200 g for 3 minutes. Cell pellets were re-suspended in 200 µl of PBS and counted using the trypan blue exclusion method. Cells were diluted in phosphate buffered saline (PBS) and 4x SDS loading buffer to give a final density of 250,000 cells per 50 µl. Cell suspensions were sonicated on ice three times for 5 seconds with 30 second intervals and stored at −80°C until used.

25 µg of total protein extract from tissue or 250,000 cells from culture experiments from each age was loaded into 12.5% acrylamide gels and resolved at 150 volts for 50 minutes. The gel was removed from the cassette and briefly rinsed in distilled water. Semi-dry blotting was used to transfer proteins to a nitrocellulose membrane over a period of 60 minutes at 10 volts. The membrane was probed with specific antibodies to glyceraldehyde 3-phosphate dehydrogenase (GAPDH; Abcam; 1∶1,000), VDR (VDR; Abcam; 1∶1,000) or glial derived neurotrophic factor (GDNF; 1∶1,000; Abcam). Membranes were first blocked using 5% bovine serum albumin (BSA) in TBST (Tris buffered saline containing 0.05% Tween 20) for 1 hour, before probing with primary antibodies diluted in TBST containing 1% non-fat milk for 1 hour, except for GAPDH where 1% BSA was substituted for 1% non-fat milk. Membranes were then washed thoroughly with TBST before incubating with appropriate horse radish peroxidase-conjugated secondary antibodies (Pierce) for 1 hour at a 1∶10,000 dilution. Membranes were again washed thoroughly with TBST before bands were detected using Pierce ECL Western blotting substrate in a dark room. Images were initially exposed for 60 seconds, and further images produced using longer or shorter exposure times depending on the intensity of the bands in the initial image. Images were documented and bands quantified using integrated optical density readings.

### Statistical Analysis

All cell counts were produced from a minimum of 6 coverslips, and Western blots from a minimum of 3 replicates. Cell counts were performed using NIS Elements version 3 and statistical interpretation performed using GraphPad version 5. Data plotted on graphs is expressed as mean ± SEM. Where more than two conditions were compared ANOVA was followed by a Bonferroni post-hoc test. Where two sets of data were compared a two tailed unpaired t-test was performed. Results were considered statistically significant for p values less than 0.05.
